# Early Detection and Analysis of Cavity Defects in Concrete Columns Based on Infrared Thermography and Finite Element Analysis

**DOI:** 10.3390/ma18071686

**Published:** 2025-04-07

**Authors:** Fan Yang, Xianwang Zeng, Qilong Xia, Ligui Yang, Haonan Cai, Chongsheng Cheng

**Affiliations:** 1 No.3 Construction Co., Ltd., Chongqing Construction Engineering Group, Chongqing 481102, China; cqwushengjiu@163.com; 2 School of Civil Engineering, Chongqing Jiaotong University, Chongqing 400074, China; 622230970107@mails.cqjtu.edu.cn (X.Z.); yangligui@cqjtu.edu.cn (L.Y.); 611210080005@mails.cqjtu.edu.cn (H.C.); 3 State Key Laboratory of Mountain Bridge and Tunnel Engineering, Chongqing Jiaotong University, Chongqing 400074, China

**Keywords:** infrared thermography, hydration heat, concrete structures, thermal contrast

## Abstract

Concrete, known for its high strength, durability, and flexibility, is a core material in construction. However, defects such as voids and honeycombing often occur due to improper pouring or vibration, weakening the concrete’s strength and affecting its long-term performance. These defects typically require costly repairs. Therefore, timely identification and repair of such early defects is crucial for improving construction quality. This paper proposes a method for non-destructive detection of honeycomb defects in concrete using infrared thermography (IR) during the hydration stage. By analyzing the temperature differences between defect and non-defect areas based on the temperature distribution generated during hydration, defects can be detected. Furthermore, the study uses the COMSOL finite element model to explore the relationship between defect size, ambient temperature, formwork thickness, and thermal contrast. The results show that IR technology can effectively and reliably detect honeycomb defects, especially during the hydration phase. As a convenient and feasible non-destructive testing method, IR technology has significant potential for application and development in concrete defect detection.

## 1. Introduction

Concrete is a versatile material used in constructing bridges, roads, buildings, and other structures, owing to its ability to be molded into complex shapes and meet diverse construction requirements. Due to its high compressive strength, durability, and superior workability, concrete has emerged as a critical component in contemporary civil engineering practices [1,2]. However, during concrete pouring, poor fluidity, insufficient vibration, and adverse environmental conditions often result in the aggregates failing to compact fully, leading to the formation of localized defects such as honeycombs and voids. These early-stage defects compromise concrete compactness, accelerating structural degradation and performance decline. More critically, they persistently undermine integrity throughout the service lifespan, posing significant safety risks [3,4]. During the concrete pouring stage, if honeycomb defects in the concrete can be promptly identified, timely remedies (such as vibration or tamping) can be applied using non-destructive methods. However, once the concrete has hardened, repairing these defects often requires significant human and material resources, thereby substantially increasing construction costs. Therefore, quickly and accurately detecting honeycomb and void defects in concrete during the pouring stage holds significant value.

In recent years, with the advancement of non-destructive testing (NDT) technologies, numerous NDT methods for detecting defects in concrete structures have emerged [5,6,7,8,9]. Zhao et al. [10] combined Digital Image Correlation (DIC) technology with wavelet analysis to identify and warn of concrete structures of micro-damage. Through four-point bending experiments and the analysis of acceleration response signals, the experimental results demonstrated that this method can effectively provide early warnings for micro-damage and accurately locate and assess the extent of the damage. Janku et al. [11] measured concrete bridges and defect test specimens using various NDT techniques, providing a comprehensive comparison in terms of accuracy, operability, and cost. Jiao et al. [12] developed an automated defect detection algorithm for concrete based on Ground Penetrating Radar (GPR). The algorithm utilizes signal polarity and morphological features to identify delamination, voids, and water infiltration defects. Experimental results showed the algorithm’s strong detection capabilities in both simulated and real-world data. McCabe et al. [13] utilized GPR as a non-destructive technique to detect early characteristics of honeycomb defects in concrete pavements. Experiments validated the sensitivity of GPR in identifying the size, shape, and depth of honeycombs, providing a reference for its application in pavement defect detection. Chow et al. [14] proposed an automated concrete defect detection framework based on a 360° camera and LiDAR. The framework utilizes deep learning algorithms to identify defects and integrates the results into a Building Information Model (BIM), effectively addressing the inefficiency of traditional manual inspection methods. Christoph et al. [15] proposed a concrete honeycomb defect detection method based on multi-sensor data fusion. By integrating impact echo (IE), Ultrasonic (US), and GPR technologies, the method significantly improved detection reliability and accuracy through a feature-level data fusion algorithm. Xu et al. [16] combined deep learning technology with the impact echo method, utilizing wavelet transform to extract signal features and establishing a deep learning network detection system. The results demonstrated that this system can efficiently and accurately identify defects in concrete structures, achieving high detection accuracy. However, these techniques still exhibit limitations in large-area coverage, adaptability to complex environments, and real-time monitoring capabilities. In particular, there is an urgent need for more efficient and reliable solutions for the emergent detection of concrete defects and evaluating their dynamic evolution during the early stage of concrete hardening.

Infrared thermography (IRT), as a non-destructive testing method based on temperature distribution differences, demonstrates unique advantages in concrete defect detection due to its portability, efficiency, non-contact nature, and large-area coverage capabilities [17]. IRT can rapidly capture temperature anomalies in infrared radiation, accurately locating defect areas, making it particularly suitable for real-time monitoring in complex environments and during hydration processes. Compared to other NDT techniques, IRT not only offers higher detection efficiency but also enables the rapid acquisition of inspection results without direct contact with the concrete structure. Cheng et al. [18] utilized IRT and elastic wave technology to scan concrete specimens with prefabricated defects and analyzed the size of the defects based on thermal images and elastic wave signals. Vemuri et al. [19] employed IRT to detect defects in concrete structures and concluded that IRT offers advantages such as short detection time and precise localization. Cheng et al. [20] cast concrete specimens with defects and utilized IRT to detect these defects during concrete hydration. Hong et al. [21] utilized IRT and Digital Image Correlation (DIC) technologies to detect early defects in concrete. The results indicated that IRT could accurately identify surface and internal voids, and the geometric dimensions of the voids significantly affected the temperature difference, showing a power function relationship with the product of thickness and depth. Cheng et al. [22] investigated the effect of different time windows on the detection performance of concrete delamination defects using infrared thermography (IRT). They found that the appropriate selection of time windows is critical for improving detection sensitivity and proposed the optimal time window for delamination detection under natural conditions.

Although IRT has unique advantages in concrete defect detection, its application is typically limited to the post-hydration stage. In this hardened state, remediation of identified defects incurs high complexity and cost. In contrast, during the hydration stage, the concrete is still malleable, and timely detection and intervention can effectively prevent defects from solidifying, significantly reducing repair costs and difficulty. Therefore, using IRT for defect detection during the hydration stage is of great significance. This study is the first to explore the use of IRT for detecting honeycomb defects during the early stages of concrete hydration. Unlike previous studies, this research focuses on the temperature changes caused by the release of hydration heat, using the temperature difference between defective and non-defective areas to detect honeycomb defects [23]. This method enables early intervention when defects occur, preventing high-cost and complex repairs later, and improving the overall quality of concrete construction. Garg et al. [24] experimentally studied the feasibility of using hydration heat as an internal heat source to detect voids in prestressed tendon ducts. The results demonstrated that this method is effective for void detection. Li et al. [25] proposed a method for detecting grouting defects in external prestressed tendon ducts using IRT during the hydration stage and discussed the impact of defect severity on the infrared detection capability. Wan et al. [26] experimentally studied the application of IRT in defect detection during the concrete hydration process, defining the detection time window and identifying the optimal timing for IRT application. Cai et al. [27] proposed enhancing the detection performance of IRT for interface debonding defects in CFST arch bridges by utilizing concrete hydration heat and water cooling. Experiments demonstrated that thermal contrast improved by 2–3 times after cooling excitation, and numerical simulations confirmed a linear relationship between cooling intensity and thermal contrast. This method shows potential for early debonding detection. Cheng et al. [28] experimentally investigated the effects of hydration heat rising rate, void size, and environmental factors on the detection of debonding in CFST. The results showed that the absolute temperature difference had the most significant impact on detection performance, while the hydration rate had the least effect. The interaction between hydration rate and void size had a secondary impact on detection performance, and this interaction weakened as both the hydration rate and void size decreased.

This study aims to conduct numerical and experimental investigations on IRT detection of honeycomb defects during the hydration stage of concrete under formwork conditions. First, an experimental model is developed using COMSOL (version 6.2) finite element simulations to investigate the feasibility and efficiency of infrared thermography (IRT) in detecting defects during the concrete hydration stage. Next, physical experiments are conducted to validate the simulation results, confirming the model’s accuracy and reliability in replicating the hydration process. Finally, the validated model is applied to analyze the effects of ambient temperature, honeycomb defect size, and formwork thickness on the thermal contrast evolution between defective and intact regions. This study proposes a concrete honeycomb defect detection method based on infrared thermography technology, combined with finite element simulation, to thoroughly investigate the impact of various factors on detection effectiveness. It provides an innovative technical approach and methodology for detecting honeycomb defects during the concrete hydration stage. Additionally, it offers significant theoretical foundations and practical references for optimizing and developing defect detection technologies under complex working conditions. Particularly, the integration of numerical simulation and physical experiments to verify and optimize the detection method ensures its reliability and applicability, providing strong technical support for practical engineering applications.

## 2. Materials and Methods

During the concrete pouring, construction issues such as insufficient vibration may result in honeycomb defects in localized areas. These defects are typically air-filled, leading to a significantly lower thermal conductivity than the surrounding dense concrete. This difference causes temperature variations between the defective and non-defective regions. By analyzing the temperature differences on the surface of the concrete formwork, early defects can be effectively identified and located. Therefore, conducting an in-depth analysis of the temperature difference (i.e., thermal contrast) between defective and non-defective regions is of significant importance for the early detection of defects.

**Figure 1:**
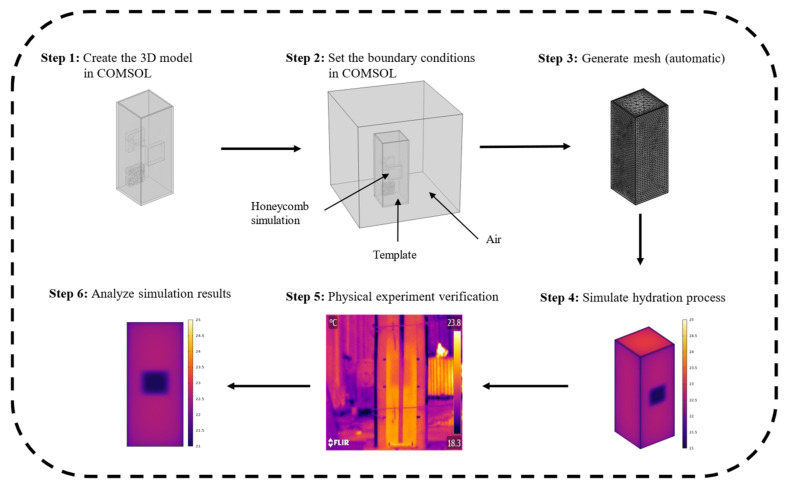
Research framework.

During the concrete hydration process, the temperature difference on the surface of the formwork is influenced by multiple factors, including ambient temperature, formwork thickness, the particle size of the honeycomb defect, and the initial pouring temperature of the concrete. As shown in Figure 1, this study employed the finite element method (COMSOL Multiphysics) to simulate the heat conduction process in concrete structures containing honeycomb defects under hydration heat conditions. The research procedure was as follows: (1) a three-dimensional concrete model with honeycomb defect was established; (2) boundary conditions were defined for the model; (3) the model was meshed according to the shape and size of the material; (4) the concrete hydration process was simulated; (5) real concrete specimens were cast to validate the 3D model and assessed its accuracy; and (6) the simulation results were analyzed to reveal the variation patterns of thermal contrast. Subsequently, a parametric study was conducted to quantify the impact of ambient temperature, formwork thickness, and defect size on the detection sensitivity of honeycomb defects during hydration.

### 2.1. Concrete Hydration Heat Calculation

Hydration heat, a fundamental phenomenon occurring during concrete hydration, induces internal temperature variations due to exothermic chemical reactions. This thermal behavior can be accurately modeled through numerical simulations, offering a robust approach to investigating spatiotemporal temperature field evolution in hydrating concrete. In this study, the hydration heat process was simulated using COMSOL Multiphysics software (version 6.2), where heat transfer was modeled as a transient solid-phase conduction process. The results quantitatively delineated the temperature field dynamics driven by hydration heat, facilitating in-depth analysis of formwork surface temperature gradients and their implications for defect detection.

Zhu [29] explicitly pointed out that cement hydration heat is closely related to the age of the concrete. In commonly used models for calculating cement hydration heat, there are typically three main types: the exponential calculation model, the composite exponential calculation model, and the hyperbolic calculation model. Liu [30] compared the three hydration heat calculation models by comparing their calculated results with the measured data. The results showed that the calculated results of the composite exponential hydration heat model were closer to the measured values. Through a series of experimental and computational studies, Lin et al. [31,32] conducted an in-depth investigation of the hydration heat release model. They concluded that, compared to other models, the composite exponential model exhibits higher accuracy in describing the hydration heat release process. In this study, the composite exponential model was adopted to calculate the cement hydration heat, as expressed in Equation (1) [33]:

(1) $$Q(t)=Q_{0}\left(1-e^{-at^{b}}\right)$$

where *Q*(*t*) is the cumulative heat of hydration of concrete at time *t*, and its unit is kJ/kg. *Q*_0_ is the cumulative hydration heat as *t*→∞, with its value selected based on reference [33], and its unit is kJ/kg. *t* is the time, and its unit is days (*d*). *a*, *b* are constants related to cement varieties.

Rewriting Equation (1) to the unit of hours and the derivative of time yields the formula for the heat generation rate of concrete, after unit conversion: 1 w = 1 J/s = 3.6 kJ/h, as (2):

(2) $$HENG=W\frac{dQ\left(t\right)}{dt}=\frac{ab}{24}WQ_{0}t^{b-1}e^{-a{\left(\frac{t}{24}\right)}^{b}}$$

where *Q*(*t*) is the cumulative heat of hydration of concrete at time *t*, and its unit is kJ/kg. *Q*_0_ is the cumulative hydration heat as *t*→∞, with its value selected based on reference [33], and its unit is kJ/kg. *t* is the time, and its unit is days (*d*). *a*, *b* are constants related to cement varieties. *W* is the cement content, and its unit is kg/m^3^. *HENG* is the heat generation rate, and its unit is kJ/(h·m^3^).

After unit conversion, 1 W = 1 J/s = 3.6 kJ/h, the heat generation rate *HENG* can be obtained as follows:

(3) $$HGEN=W\frac{dQ\left(t\right)}{dt}=\frac{\frac{ab}{24}WQ_{0}t^{b-1}e^{-a{\left(\frac{t}{24}\right)}^{b}}}{3.6}=\frac{abWQ_{0}t^{b-1}e^{-a{\left(\frac{t}{24}\right)}^{b}}}{24\times 3.6}$$

### 2.2. Numerical Modeling

**Figure 2:**
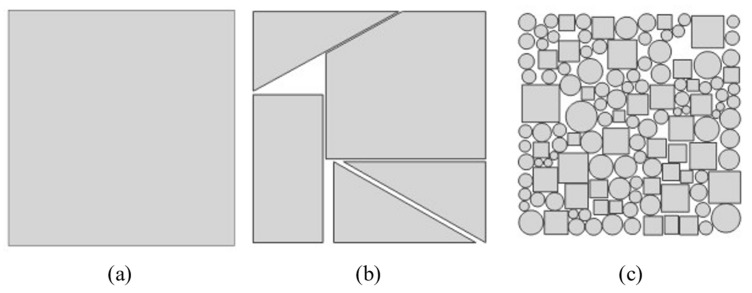
Honeycomb defect model design: (**a**) 200 mm; (**b**) 40–50 mm; (**c**) 10–20 mm.

This section focuses on the establishment of the finite element model. The accurate simulation of honeycomb defects forms the basis for temperature field analysis during the concrete hydration process, as the presence of honeycomb defects significantly affects the temperature field distribution on the surface of concrete formwork. To precisely simulate the detection of honeycomb defects, a concrete column model with dimensions of 400 mm × 400 mm × 1000 mm was designed. A honeycomb defect region measuring 200 mm × 200 mm was placed at the center of the inner surface of the formwork, with these defects typically filled with air. Additionally, to further investigate the sensitivity of infrared thermography to honeycomb defects with varying particle sizes, three types of honeycomb defects were designed: defects with particle sizes of 10–20 mm, 40–50 mm, and a single defect block with an overall size of 200 mm × 200 mm. The specific layout of the defects is shown in Figure 2.

**Figure 3:**
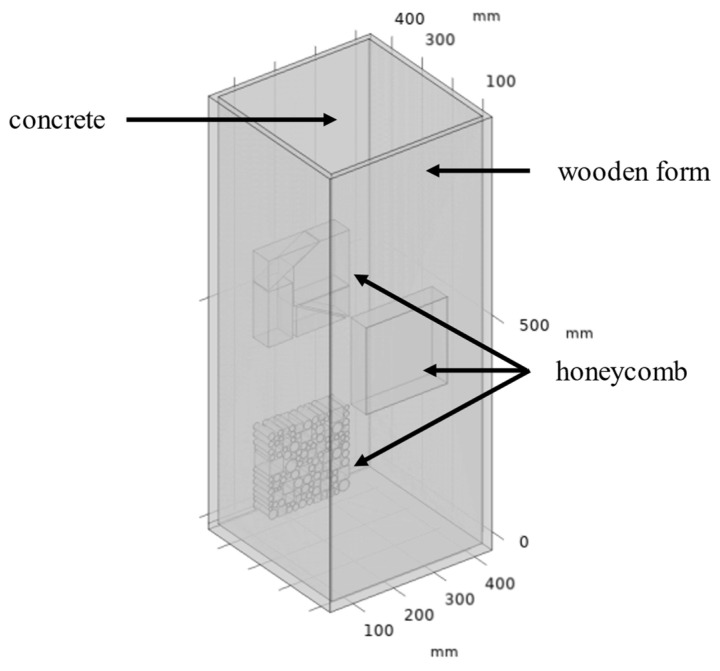
Geometric shape of the concrete column model.

**Figure 4:**
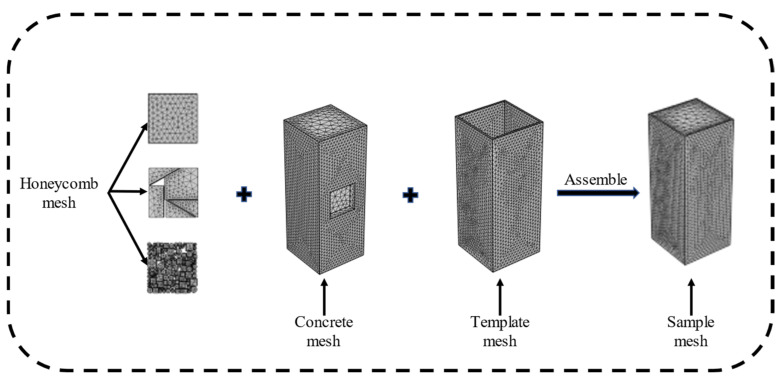
Mesh division of the concrete column model.

To study the detection of honeycomb defects, a three-dimensional finite element model (as shown in Figure 3) was developed using the heat transfer module in COMSOL Multiphysics software to simulate the concrete hydration process. The material parameters used in the model are listed in Table 1. In this model, the formwork, concrete column, and honeycomb defect regions were finely meshed to improve computational accuracy (as shown in Figure 4). The meshing used a “physics-controlled mesh” setting, with the element size set to “finer”, resulting in a total of 135,201 domain elements, 32,656 boundary elements, and 4405 edge elements.

In the numerical simulation, natural convection boundary conditions were applied at the formwork-air interface to represent the heat exchange process. Fresh concrete has high fluidity and self-weight characteristics. During the pouring stage, the concrete-formwork system formed a gapless contact interface. As a result, the impact of interfacial contact thermal resistance on the heat transfer process was neglected in the model. To capture the transient nature of the heat released during hydration, an unsteady-state heat transfer model with a time step of 1 min was established. This setting ensured the accurate representation of the spatial and temporal evolution of the temperature field on the formwork surface while balancing computational accuracy and efficiency. By adopting physically justified boundary conditions and dynamic solution strategies, the study successfully simulated the temperature difference formation mechanism on the formwork surface during hydration. Furthermore, the mapping relationship between these temperature differences and defect features was quantitatively characterized.

### 2.3. Case Design

This study specifically examined the impact of honeycomb size, ambient temperature, and formwork dimensions on detecting honeycomb defects in concrete structures. The study designed a 200 mm × 200 mm area as the honeycomb simulation region. Apart from differences in particle size, the honeycomb particles occupied 50% of the total simulation area. Additionally, a single 200 mm × 200 mm solid honeycomb block was designed to simulate a large void in the concrete structure. The relevant working condition information is shown in Table 2. Specifically, the defect sizes were determined based on common types of concrete defects in actual engineering projects: 200×200 mm corresponds to void defects, and 10–30 mm and 30–60 mm correspond to small and large honeycomb defects, respectively, while 4–10 mm corresponds to surface pitting defects. For formwork thickness, considering that the most commonly used formwork thickness in engineering practice falls within the 10–20 mm range [34], the study selected several representative thickness values within this range for comparative analysis to investigate the effect of formwork thickness on detection performance.

### 2.4. Evaluation Indicators

**Figure 5:**
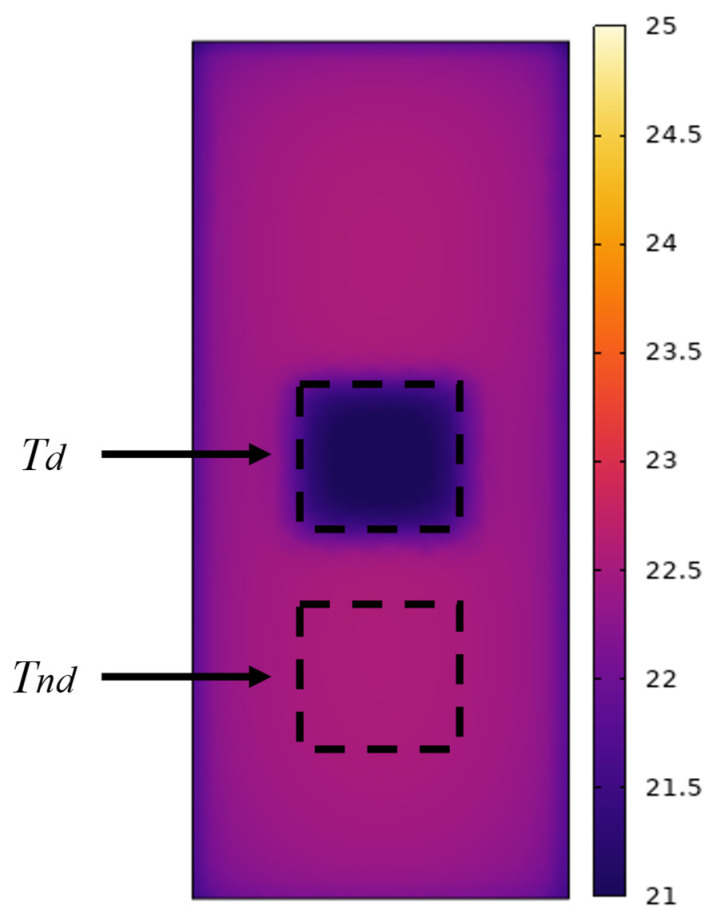
Schematic diagram of infrared thermal contrast calculation.

When honeycomb defects exist in a concrete structure, air entrapped in the defect area results in a significant thermal conductivity contrast between the concrete and air, creating a measurable temperature gradient between the two regions. This study analyzed the temperature difference between the honeycomb defect area and the non-defect area as an indicator of detection performance. As shown in Figure 5, the temperature difference (also referred to as absolute thermal contrast) can be defined using the following equation:

(4) ΔT=Tnd−Td

in the equation, *Tnd*, *Td*, and Δ*T* represent the average surface temperature of the non-defect area, the defect area, and the temperature difference between the non-defect and defect areas, respectively. When Δ*T* > 0, it indicates that *Tnd* > *Td*, meaning the temperature of the non-defect area is higher than that of the defect area. Conversely, when Δ*T* < 0, it indicates that the temperature of the non-defect area is lower than that of the defect area. To enhance the clarity and coherence of the paper, several parameters were defined for use in subsequent analysis and discussion. The details are provided in Table 3.

## 3. Experimental Validation

### 3.1. Experimental Design

**Figure 6:**
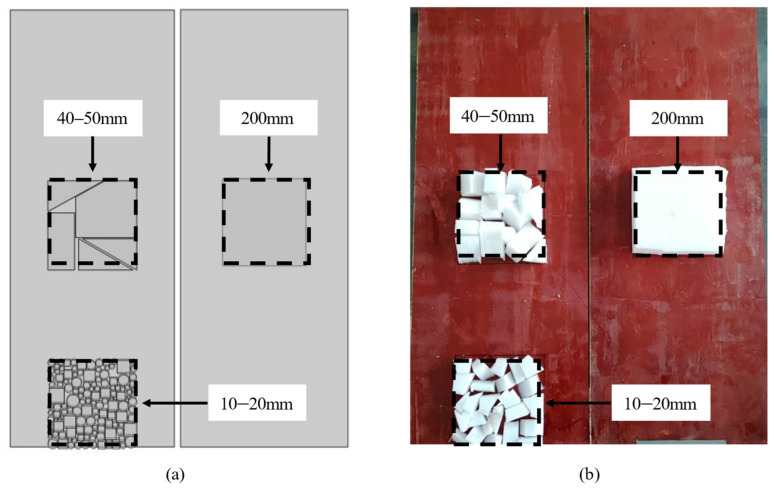
Honeycomb defect design diagram: (**a**) finite element simulation defect layout; (**b**) physical experiment defect layout.

**Figure 7:**
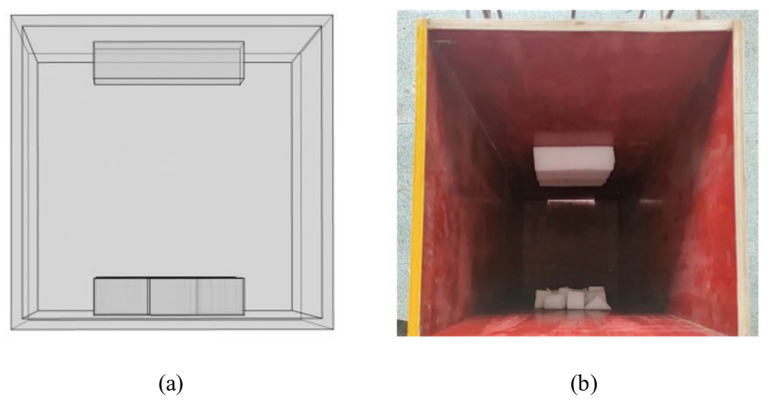
Concrete formwork frame design: (**a**) finite element simulation formwork frame; (**b**) physical experiment formwork frame.

In the physical experiment, three honeycomb defects identical to those in the simulation were set up, and their spatial configuration is shown in Figure 6. After assembling the formwork containing pre-installed defects, a formwork system enclosing the concrete was assembled. The detailed structure and arrangement of the formwork frame are shown in Figure 7.

**Figure 8:**
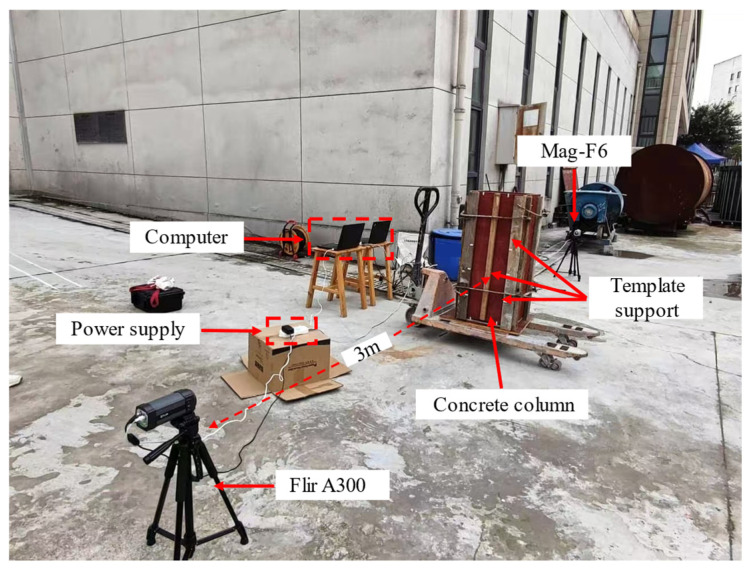
Experimental site layout.

This study used the FLIR A300 and MAG-F6 infrared thermal imaging cameras to perform infrared detection on the experimental specimens. The parameters of the thermal imaging cameras are listed in Table 4. The FLIR A300 camera has a resolution of 320 × 240 pixels, a spectral range of 7.5–13 μm, and an accuracy of ±2 °C or ±2%. The MAG-F6 camera has a resolution of 640 × 480 pixels, a spectral range of 7.5–13 μm, and an accuracy of ±0.7 °C or ±0.7%. Both cameras were positioned on the formwork side adjacent to the honeycomb defect, approximately 3 m from the outer surface of the specimen, with their height aligned with the center height of the specimen. Data acquisition was configured at a sampling rate of 1 frame/min, and the thermal images were stored on a connected PC. The detailed layout of the experimental site is shown in Figure 8.

As shown in Figure 8, the formwork was reinforced to ensure the smooth progress of the experiment and prevent the formwork from being damaged due to excessive force. Before the experiment officially began, the infrared data acquisition equipment (computer) was used to preset the acquisition frequency of the infrared thermal camera. Parameters affecting the accuracy of temperature measurements, such as measurement distance, humidity, airflow velocity, and emissivity, were also configured to ensure the precision of infrared temperature measurement. The height and focus of the cameras were precisely adjusted to ensure that the infrared image acquisition system obtains clear and accurate images. Subsequently, the temperature data acquisition system was activated simultaneously with the concrete pouring process to obtain real-time temperature distribution images of the column specimen’s surface.

**Figure 9:**
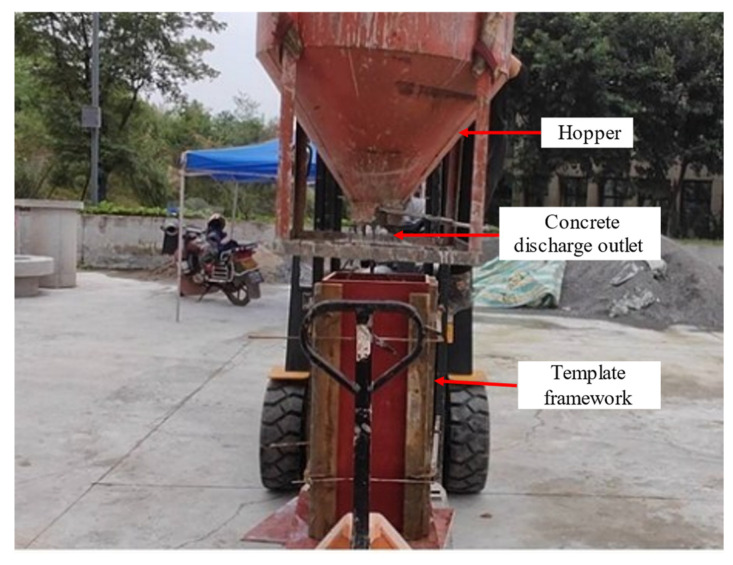
On-site record of concrete pouring.

The concrete pouring process is shown in Figure 9. First, the uniformly mixed concrete was loaded into a hopper, which was then moved above the concrete column structure using a forklift. Next, the hopper valve was opened to pour concrete until the formwork was fully filled. The entire process was carried out strictly by the experimental design requirements to ensure the accuracy of data collection.

### 3.2. Experimental Materials

This study designed physical specimens with dimensions consistent with the simulation model and used experimental data to validate the numerical model. The materials required for producing concrete include cement, sand, water, and aggregates. The concrete mix design followed the “*Technical Specification for High-Strength Concrete Structures*” (CECS104:99). The material mix proportion for the concrete specimen is shown in Table 5. The sand (fine aggregates) had a maximum particle size of 2.36 mm, while the crushed stone (coarse aggregates) ranged from 4.75 mm to 9.5 mm.

In actual concrete pouring processes, honeycomb defects (air voids) typically form due to insufficient vibration, and these honeycomb defects are filled with air. Under standard atmospheric pressure at 15 °C, the thermal conductivity values are as follows: air (0.023 W/(m·K)), concrete (1.7 W/(m·K)), and polystyrene foam (0.02–0.05 W/(m·K)). The thermal conductivity of polyethylene foam is close to that of air; therefore, this experiment used polystyrene foam as the prefabricated material to simulate a honeycomb defect. The formwork for casting the concrete columns was constructed from standard wooden formwork, with thickness dimensions matching those defined in the simulation model.

### 3.3. Validation Results

**Figure 10:**
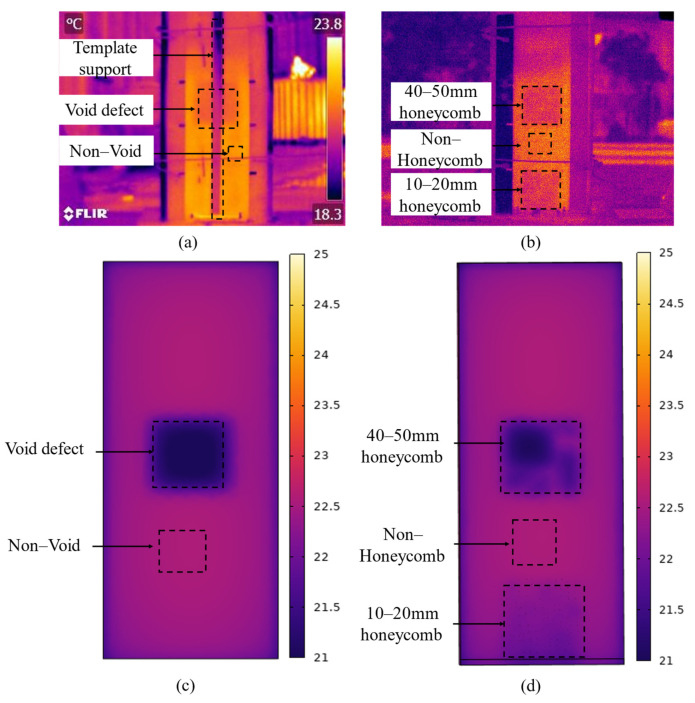
Comparison of infrared images of honeycomb defect: (**a**,**b**) results from physical experiments; (**c**,**d**) results from finite element simulations.

As shown in Figure 10, five minutes after pouring, the defects began to appear. Additionally, a vertical shadow region appeared at the column center in the infrared image. This shadow resulted from the formwork frame’s support structure, which physically blocked the infrared detection of the honeycomb defect region. However, excluding this elongated shadow region, significant temperature differences were still observed between the defect and non-defect areas in Figure 10a,b. At this stage, the thermal contrast (Δ*T*) between the non-defect area and the defect area was 0.2 °C. Thus, a thermal contrast threshold of 0.2 °C was established in this study. The results demonstrate that infrared thermography enables real-time detection of honeycomb defects in early-stage concrete hydration.

To validate the accuracy of the finite element (FE) simulations for concrete structures, experimental infrared thermograms were compared with FE-simulated temperature distributions. Figure 10c,d shows the infrared temperature images obtained by simulating the concrete hydration process using COMSOL Multiphysics software. To ensure the consistency of the validation results, the temperature data from both the experimental and simulated images were extracted using the same method. Specifically, the honeycomb defect area’s average temperature was selected as the honeycomb defect’s surface temperature, while the surrounding area’s average temperature was selected as the surface temperature of the non-defective area. Through comparative analysis, the consistency between the simulation results and the experimental data were validated, further demonstrating the reliability of the simulation model.

**Figure 11:**
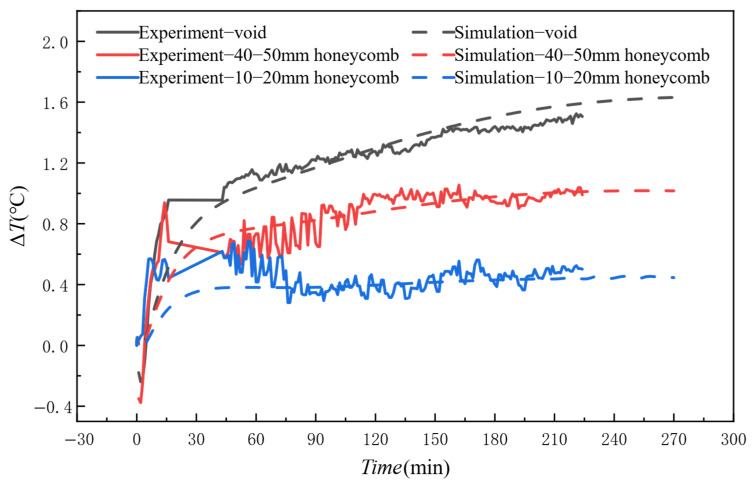
Comparative analysis of experimental and simulated temperature data.

Figure 11 presents a detailed comparison of thermal contrast between the FE simulations and concrete experiments. After concrete pouring, the thermal contrast gradually increased over time. After 30 min, differences in thermal contrast began to emerge for different sizes. The figure shows that the larger the size of the honeycomb defect, the greater the thermal contrast Δ*T*. Experimental and simulated temperature profiles showed congruent trends, though minor deviations were observed. The mean errors between experimental and simulated curves were 0.06 °C, 0.04 °C, and 0.05 °C, respectively. These discrepancies are mainly due to three influencing factors: environmental variables, measurement system errors, and differences in simulation settings. External factors such as ambient temperature, humidity, and airflow can affect the temperature measurement accuracy of the infrared thermal camera. Furthermore, the materials used in the specimen and the idealized conditions set in the simulation model, including material properties and boundary conditions, can also introduce errors. Although there are some minor differences, these discrepancies are within an acceptable range for this study. Therefore, the simulation method used in this research demonstrates accuracy and reliability in predicting the temperature field of honeycomb defects in concrete.

## 4. Results and Analysis

### 4.1. Influence of Ambient Temperature on Thermal Contrast

This section discusses the influence of honeycomb defects under conditions where the initial concrete pouring temperature is 22.5 °C, the formwork thickness is 14 mm, the honeycomb defect size ranges from 30 mm to 60 mm, and the defect thickness is 60 mm. The analysis was conducted for ambient temperatures of 10 °C, 16 °C, 22 °C, 28 °C, and 34 °C.

**Figure 12:**
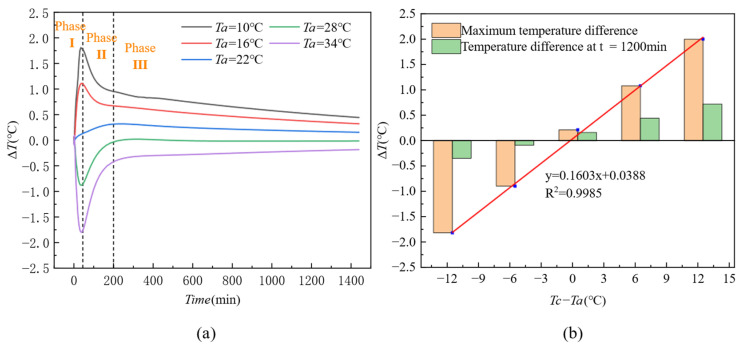
Influence of ambient temperature on thermal contrast: (**a**) variation in thermal contrast during the hydration process; (**b**) relationship between thermal contrast and the temperature difference between the initial concrete temperature and ambient temperature.

Figure 12a illustrates the trend of thermal contrast (Δ*T*) in the area of the honeycomb defect over time at different ambient temperatures (*Ta*). From the figure, it can be observed that after concrete pouring, when Ta is 10 °C and 16 °C (below the initial concrete temperature, *Tc*), Δ*T* increases sharply and reaches its peak at around 50 min (Phase I), then starts to decrease (Phase II), gradually slowing down after 200 min (Phase III). When *Ta* is 28 °C and 34 °C (above the initial concrete temperature, *Tc*), Δ*T* increases immediately in the negative direction, reaches its peak in the negative direction at around 50 min, then starts to increase again, gradually rising after 200 min. When *Ta* is 22 °C, ΔT increases slowly, reaching its peak at *t* = 200 min, and then slowly decreases and stabilizes. Based on the above analysis, it can be concluded that the greater the temperature difference between the initial concrete temperature (*Tc*) and the ambient temperature (*Ta*), the higher the peak value of Δ*T*. When the ambient temperature is closer to the initial concrete temperature, the peak value of Δ*T* is smaller. Therefore, it can be concluded that when the temperature difference (*Tc* − *Ta*) is large, Δ*T* is mainly influenced by the ambient temperature, while when *Tc* − *Ta* is small, Δ*T* is mainly influenced by the heat generated by the concrete hydration process.

Figure 12b presents bar charts of the thermal contrast at the maximum value and *t* = 1200 min for different values of *Tc* − *Ta*. It can be observed from the figure that thermal contrast is positively correlated with *Tc* − *Ta*. By fitting the relationship between the maximum thermal contrast and *Tc* − *Ta*, the following equation is obtained:

$$\begin{array}{l} y=0.1603x+0.0388\\ R^{2}=0.9985 \end{array}$$

**Figure 13:**
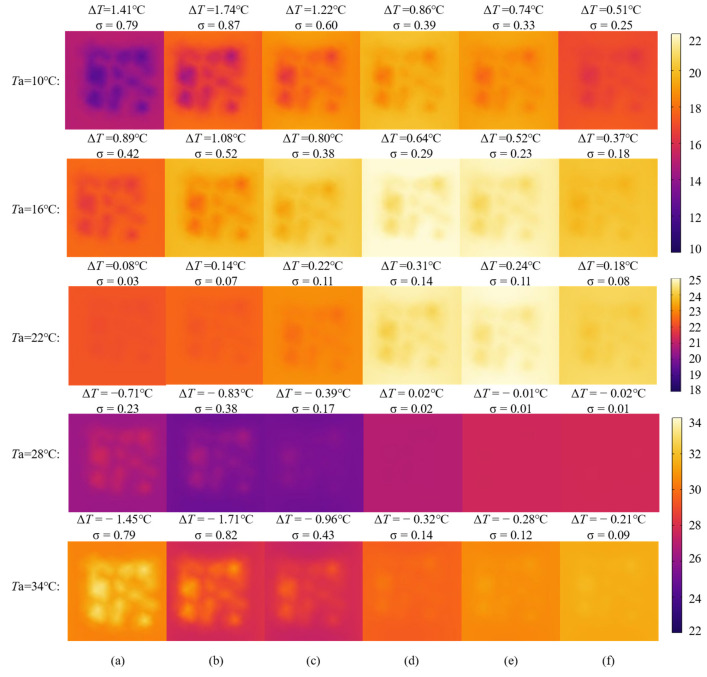
Evolution of infrared images of the honeycomb defect under different ambient temperatures: (**a**) *t* = 20 min; (**b**) *t* = 50 min; (**c**) *t* = 100 min; (**d**) *t* = 300 min; (**e**) *t* = 600 min; (**f**) *t* = 1200 min.

Figure 13 illustrates the evolution patterns of infrared images of honeycomb defects under different ambient temperatures (*Ta*). Firstly, due to the wide range of ambient temperatures, the color scale on the right is adjusted accordingly: *Ta* = 10 °C and *Ta* = 16 °C use the first color scale on the right, *Ta* = 22 °C uses the second color scale on the right, and *Ta* = 28 °C and *Ta* = 34 °C use the third color scale on the right. Secondly, at ambient temperatures of 10 °C and 16 °C, the overall trend in the infrared images shows an initial temperature rise followed by a decline. The temperature in the defect area is lower than that in the non-defect area, with the thermal contrast being greater than zero. The defect features are more pronounced in the early stages of the hydration process, but as the hydration reaction progresses, the thermal contrast gradually weakens. Additionally, the lower the ambient temperature, the greater the color contrast in the infrared images of the defect area, resulting in clearer defect features. When the ambient temperature is 22 °C, the overall changes in the infrared images are relatively minor, and the temperature distribution tends to stabilize. As the hydration reaction progresses, the color contrast between the defect and non-defect areas slightly increases. However, the overall thermal contrast remains low, making the defect features relatively indistinct. At ambient temperatures of 28 °C and 34 °C, the infrared images show an initial temperature decrease followed by an increase. The temperature in the defect area is higher than that in the non-defect area, with the thermal contrast being less than zero. In the early stages of the hydration process, the defect features are more pronounced, but as the thermal contrast weakens in the later stages, the defect features gradually become indistinct. Additionally, the higher the ambient temperature, the greater the color contrast in the infrared images of the defect area, resulting in clearer defect features. Finally, at the same hydration time, the greater the difference between the initial concrete temperature and the ambient temperature, the more pronounced the thermal contrast between the defect area and the non-defect area, resulting in clearer defect features in the infrared images.

Simultaneously, statistical analysis of temperature data is conducted for each infrared image, with the standard deviation (σ) calculated for each image. As Figure 13 demonstrates, a larger σ value indicates a more uneven distribution of temperature data across the image. Furthermore, the standard deviation exhibits a highly synchronized relationship with the absolute value of thermal contrast (|Δ*T*|), revealing their synergistic role in defect detection. These findings validate the accuracy and reliability of the selected evaluation metric (Δ*T*) proposed in this study. Consistent conclusions are confirmed in subsequent analyses presented in Section 4.2 and Section 4.3, reinforcing the robustness of the methodology.

**Figure 14:**
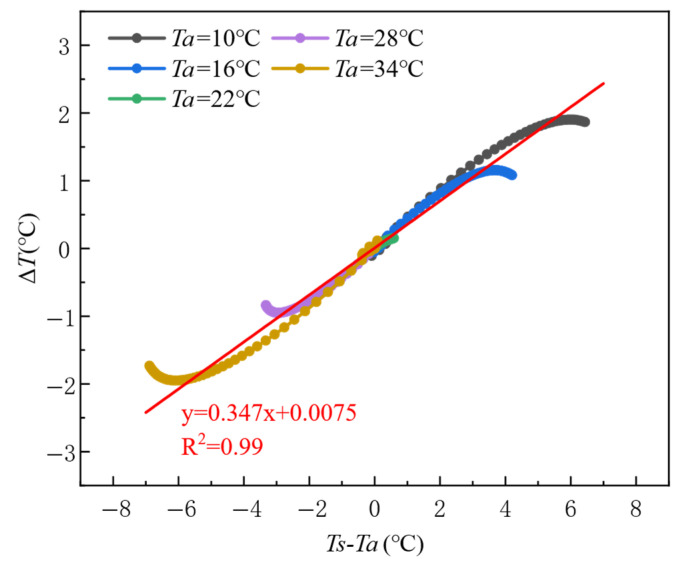
Response characteristics of thermal contrast (Δ*T*) to surface-to-ambient temperature gradient (*Ts* − *Ta*) under varying ambient temperatures during the initial casting phase (Phase I).

As evidenced by the temporal evolution characteristics in Figure 12, the study reveals that 50 min post-casting constitutes a critical threshold for thermal contrast development, during which the thermal contrast (Δ*T*) approaches its peak values across varying ambient temperature conditions. Figure 14 further elucidates the quantitative heat transfer within this 50 min window, demonstrating that the temperature gradient between the formwork surface and ambient environment (*Ts* − *Ta*) serves as the governing parameter for Δ*T* evolution. A statistically significant linear correlation emerges between these variables (y = 0.347x + 0.0075, R^2^ = 0.99). Specifically, as *Ts* − *Ta* increases from −6 °C to 6 °C, Δ*T* transitions linearly from negative to positive thermal contrast regimes, achieving peak magnitudes of approximately ±2 °C. The positive temperature gradient domain (*Ts* − *Ta* > 0) exhibits a maximum Δ*T* of +2 °C, while the negative gradient regime (*Ts* − *Ta* < 0) reaches a contrasting minimum of −2 °C.

### 4.2. Influence of Honeycomb Particle Size on Thermal Contrast

This section discusses the relationship between the thermal contrast of the area of the honeycomb defect and the non-defect area under the conditions of an initial concrete pouring temperature of 22.5 °C, a formwork thickness of 14 mm, and an ambient temperature of 16 °C. The honeycomb defect sizes considered are 4–10 mm, 10–30 mm, 30–60 mm (with a thickness of 30 mm), 30–60 mm (with a thickness of 60 mm), and a 200 × 200 mm void (with a thickness of 60 mm).

**Figure 15:**
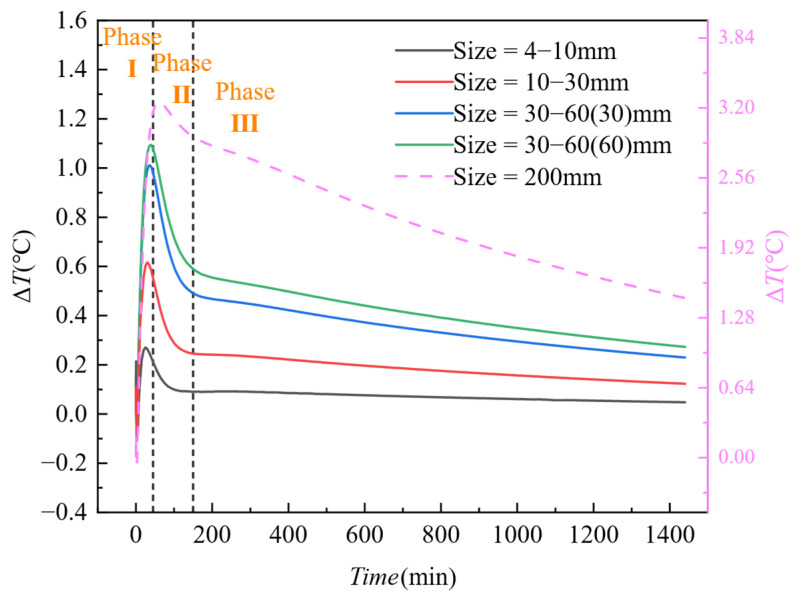
Thermal contrast trends of honeycomb defects in concrete columns under different honeycomb defect sizes.

Figure 15 shows the variation trend of thermal contrast (Δ*T*) over time for honeycomb defects of different sizes. From the figure, it can be observed that, in the early stages after concrete pouring, the thermal contrast of honeycomb defects of different sizes initially increases sharply, then reaches a peak (Phase I) and rapidly decreases (Phase II). After 200 min, the thermal contrast of the honeycomb defect starts to decrease slowly and eventually levels off (Phase III). At the same time point, the larger the size of the honeycomb defect, the higher its thermal contrast. Among them, void defects should be observed using the data on the right y-axis. It can be seen that the thermal contrast of the void defect is significantly higher than that of other types of honeycomb defects, indicating that the surface temperature of the formwork in the void defect areas reflects the differences in heat conduction more noticeably than in other defect areas.

**Figure 16:**
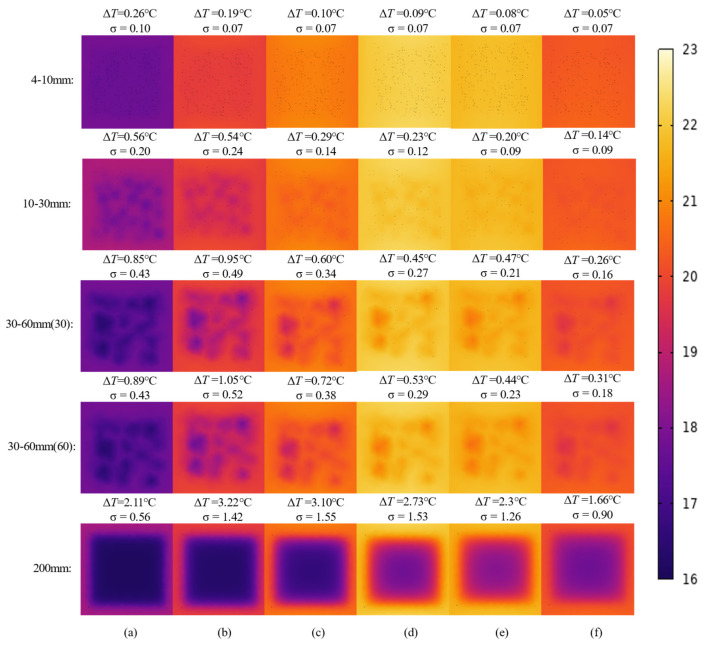
Infrared image evolution of the honeycomb defect with different honeycomb defect sizes: (**a**) *t* = 20 min; (**b**) *t* = 50 min; (**c**) *t* = 100 min; (**d**) *t* = 300 min; (**e**) *t* = 600 min; (**f**) *t* = 1200 min.

Figure 16 illustrates the evolution pattern of infrared images of honeycomb defects with different sizes. Overall, the colors in the figure transition gradually from deep purple to yellow, reflecting the gradual increase in the formwork surface temperature, followed by a subsequent decrease. This temperature variation is primarily influenced by the differences in heat transfer between the defect and non-defect areas, as the presence of a honeycomb defect significantly alters the temperature distribution characteristics of the formwork surface. Additionally, honeycomb defects of different sizes exhibit noticeable differences in their thermal behavior. For smaller honeycomb defects (4–10 mm, 10–30 mm), during the hydration process, the color contrast in the defect areas of the infrared images is minimal, especially for defects in the 4–10 mm range, where only a slight color difference is visible in the early stages. For defects in the 10–30 mm range, the color contrast in the infrared images is slightly more noticeable compared to the 4–10 mm defects. For medium-sized honeycomb defects (30–60 mm), throughout the entire hydration stage, the color contrast in the defect areas of the infrared images is higher than that of small-sized defects, indicating a greater temperature variation between the defect and non-defect areas. Additionally, the defect remains visible in the infrared images for a longer period. For void defects (200 mm), throughout the entire hydration process, the color contrast in the defect areas of the infrared images is consistently significantly higher than that of defects of other sizes. This indicates that void defects strongly impede heat transfer, resulting in a pronounced increase in color contrast between defect and non-defect areas, with the thermal contrast being the most prominent. Finally, at the same point in time, the larger the size of the honeycomb defect, the more pronounced the thermal contrast between the defect area and the non-defect area, and the clearer the characteristics of the defect area appear in the infrared images.

**Figure 17:**
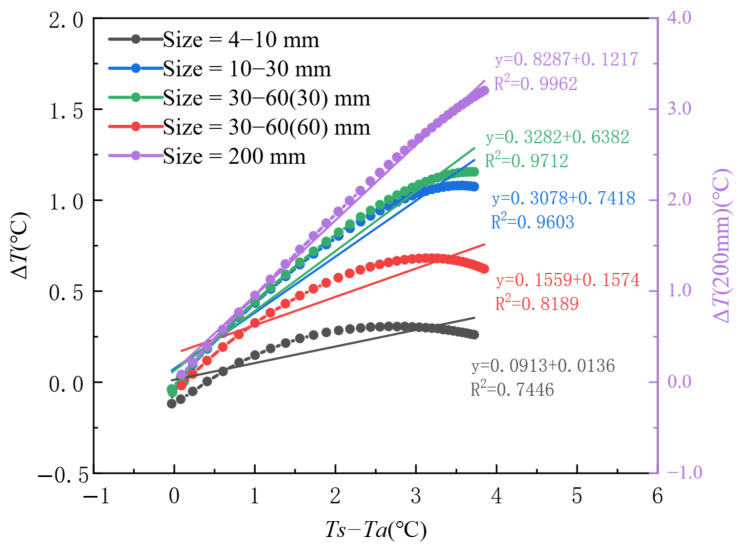
Response characteristics of thermal contrast (Δ*T*) to surface-to-ambient temperature gradient (*Ts* − *Ta*) under varying defect sizes during the initial casting phase (Phase I).

As evidenced by the temporal evolution characteristics in Figure 15, the critical time threshold for thermal contrast development is identified at 45 min post-casting, during which the thermal contrast Δ*T* reaches peak responses across varying honeycomb defect sizes. Figure 17 further elucidates the coupled size-gradient mechanism governing heat transfer: When the formwork surface-to-ambient temperature gradient (*Ts* − *Ta*) exceeds 2 °C, the fitted slope increases ninefold from 0.0913 to 0.8287 as defect size expands from 4 to 10 mm to 200 mm, confirming enhanced linear sensitivity in large-scale defects. Notably, sublinear growth (*R*^2^ = 0.74–0.82) dominates small defects (<30 mm) under *Ts* − *Ta* ∈ [0, 3] °C, whereas large defects (>30 mm) exhibit robust linearity (*R*^2^ > 0.96) within the same gradient range. Dual-axis scaling analysis reveals a 12-fold amplification in peak Δ*T* (3.2 °C for 200 mm defects vs. 0.26 °C for 4–10 mm defects), demonstrating that defect size governs thermal contrast amplification effects.

### 4.3. Influence of Plate Thickness on Thermal Contrast

This section discusses the relationship between the thermal contrast of honeycomb defect areas and non-defect areas under different formwork thicknesses. The study was conducted under the conditions of an initial concrete pouring temperature of 22.5 °C, an ambient temperature of 16 °C, and honeycomb particle sizes of 30–60 mm (with a thickness of 60 mm).

**Figure 18:**
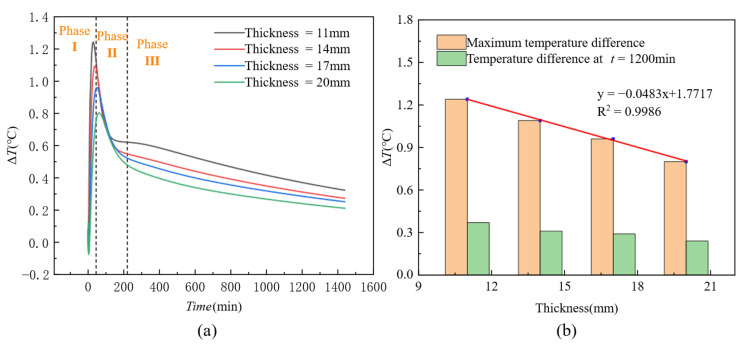
Influence of formwork thicknesses on thermal contrast: (**a**) variation in thermal contrast with the hydration process; (**b**) relationship between thermal contrast and formwork thickness.

Figure 18 illustrates the trend of the thermal contrast (Δ*T*) of honeycomb defects over time under different formwork thicknesses. As shown in Figure 18a, after concrete pouring, the thermal contrast (Δ*T*) for formwork of different thicknesses initially increases sharply and reaches a peak (Phase I), followed by a rapid decrease (Phase II). After approximately 200 min, all curves begin to decrease gradually (Phase III), with the trends of the Δ*T* curves for formworks of different thicknesses becoming essentially similar. Furthermore, it is observed that the smaller the formwork thickness, the higher the peak value and the greater the final thermal contrast. Conversely, the larger the formwork thickness, the lower the peak value and the smaller the final thermal contrast. In Figure 18b, bar charts representing the maximum thermal contrast and the thermal contrast at *t* = 1200 min for different formwork thicknesses are presented. The figure demonstrates an inverse relationship between thermal contrast and formwork thickness. By fitting the relationship between maximum thermal contrast and formwork thickness, the following equation is obtained:

$$\begin{array}{l} y=-0.0483+1.7717\\ R^{2}=0.9986 \end{array}$$

**Figure 19:**
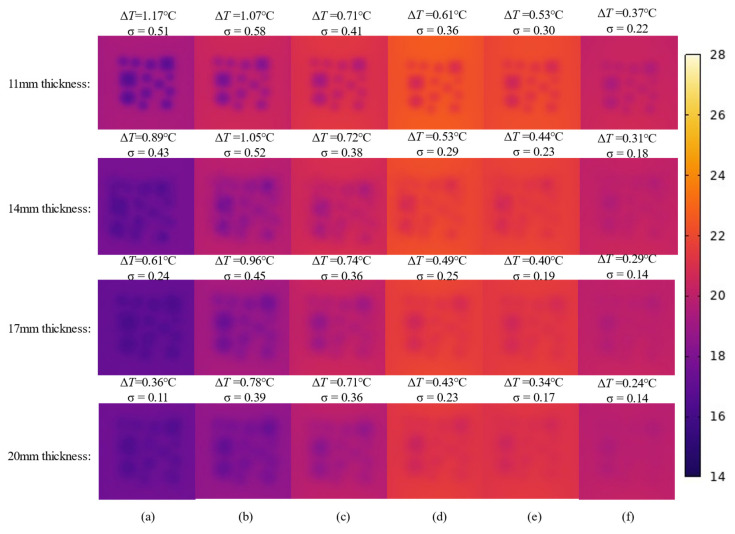
Evolution of infrared images of honeycomb defects under different formwork thicknesses: (**a**) *t* = 20 min; (**b**) *t* = 50 min; (**c**) *t* = 100 min; (**d**) *t* = 300 min; (**e**) *t* = 600 min; (**f**) *t* = 1200 min.

Figure 19 illustrates the simulated infrared temperature images at different times under various formwork thickness conditions. Overall, the colors in the figure transition from deep purple to light yellow and then back to a deeper shade, reflecting the gradual increase in the formwork surface temperature initially, followed by a subsequent decrease. The yellow areas correspond to higher surface temperatures of the formwork, while the deep purple areas correspond to lower temperatures. Secondly, for formworks with smaller thicknesses, the color contrast in the defect areas of the infrared images is greater, indicating that thinner formworks pose less resistance to heat transfer, resulting in more pronounced thermal contrast and making the defect areas easier to observe. In contrast, for thicker formworks, the color contrast in the defect areas of the infrared images is smaller, suggesting that thicker formworks provide stronger resistance to heat transfer, resulting in less pronounced thermal contrast and making the defect areas harder to detect.

**Figure 20:**
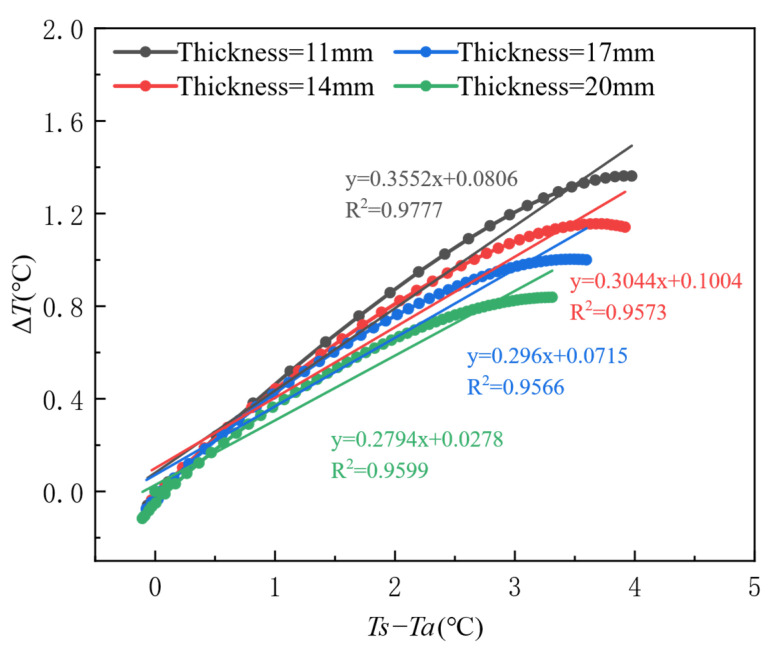
Response characteristics of thermal contrast (Δ*T*) to surface-to-ambient temperature gradient (*Ts* − *Ta*) under varying formwork thicknesses during the initial casting phase (Phase I).

Temporal evolution analysis in Figure 18 reveals that 40 min post-concrete casting marks the critical time threshold for thermal contrast development, during which the thermal contrast Δ*T* reaches peak responses across varying formwork thickness conditions. Figure 20 further quantifies the inverse correlation between formwork thickness and heat transfer efficiency: Under a constant surface-to-ambient temperature gradient (*Ts* − *Ta*) of 3 °C, increasing formwork thickness from 11 mm to 20 mm reduces the thermal contrast sensitivity coefficient (slope of linear fit) by 21.4% (from 0.3552 to 0.2794), demonstrating that thickened formwork significantly impedes thermal flux transmission and thereby diminishes defect detection sensitivity. Furthermore, all thickness formworks exhibit robust linear responses within *Ts* − *Ta* ∈ [0, 3.5] °C (*R*^2^ = 0.9566 − 0.9777).

### 4.4. Comprehensive Analysis and Technical Validation

#### 4.4.1. Influence of Key Parameters on Thermal Contrast

Based on the experimental data analysis, the control mechanisms of thermal contrast (Δ*T*) by ambient temperature (*Ta*), defect size (D), and formwork thickness (L) can be summarized as follows:

1. Ambient Temperature: The extreme response of Δ*T* is strictly controlled by the temperature gradient between the formwork surface and the environment (*Ts* − *Ta*). When the absolute value of the temperature gradient exceeds 4 °C (|*Ts* − *Ta*| > 4 °C), the peak value of Δ*T* can reach ±2 °C (*R*^2^ = 0.99). Under low-temperature conditions (*Ta* < *Tc*), Δ*T* is primarily dominated by heat dissipation from the environment (*Tc* − *Ta* > 0), whereas under high-temperature conditions (*Ta* > *Tc*), the accumulation of hydration heat takes over (*Tc* − *Ta* < 0).
2. Defect Size: The sensitivity of Δ*T* exhibits a nonlinear increase with defect size. When D ≥ 30 mm, the sensitivity coefficient (k) increases sharply from 0.0913 to 0.8287 (a 9-fold increase), with the peak value of Δ*T* reaching 3.2 °C (12 times higher than for D = 4–10 mm). Large defects (D ≥ 30 mm) show a strong linear response within the temperature gradient range of *Ts* − *Ta* ∈ [1, 4] °C (R^2^ > 0.96), while small defects (D < 30 mm) exhibit a significantly reduced linear response (*R*^2^ = 0.74–0.82).
3. Formwork Thickness: The detection sensitivity of Δ*T* is negatively correlated with formwork thickness. As the thickness increases from 11 mm to 20 mm, the sensitivity coefficient (k) decreases from 0.3552 to 0.2794 (a 21.4% reduction).
  In summary, the temperature gradient between the formwork surface and the environment is the primary driving force of Δ*T*. The defect size determines the thermal signal strength of both the defect and non-defect areas, while the formwork thickness governs the attenuation of detection sensitivity. Together, these three factors define the technical boundary for infrared detection of concrete honeycomb defects during the initial casting phase.

#### 4.4.2. Analysis of the Optimal Detection Window Based on Temporal Evolution

The time-varying characteristics of thermal contrast (Δ*T*) reveal the key time constraints for infrared detection. As shown in Figure 12a and Figure 15, and Figure 18, the evolution of Δ*T* under different conditions exhibits a three-stage pattern: “rapid rise—peak maintenance—slow decay”. The time threshold analysis is as follows:

1. Effect of Ambient Temperature: Under low-temperature (*Ta* ≤ 16 °C) and high-temperature (*Ta* ≥ 28 °C) conditions, Δ*T* reaches positive (+2.1 °C) and negative (−1.8 °C) peak values 50 ± 5 min after pouring. When the ambient temperature is close to the initial concrete temperature (*Tc*), the peak value is delayed to around 200 min.
2. Effect of Defect Size: For small defects (D < 30 mm), the ΔT peak occurs earlier, between 30 and 35 min, while for large defects (D ≥ 30 mm), the peak is delayed to 50 to 60 min.
3. Effect of Formwork Thickness: As the formwork thickness increases, the time to reach the Δ*T* peak is delayed. For example, with a formwork thickness of 11 mm, the peak occurs at 35 min, while with a thickness of 20 mm, the peak is delayed to 65 min.

In conclusion, considering the combined temporal characteristics of various factors, the optimal window for infrared detection of concrete honeycomb defects is between 35 and 60 min after pouring. During this period, the thermal contrast is close to its peak across different conditions, making it the most favorable phase for defect detection.

#### 4.4.3. Error and Uncertainty Analysis and Future Research Directions

This study demonstrates the theoretical feasibility of using infrared detection technology for identifying concrete defects through idealized models. However, its practical application still faces the following key limitations:

1. Incomplete Decoupling of Environmental Coupling Effects: In actual engineering sites, environmental conditions are complex and variable. High wind speeds can cause significant convective heat loss, which in turn affects the accuracy of temperature measurements by infrared devices and reduces the thermal contrast (Δ*T*). In addition, fluctuations in solar radiation may induce extra temperature variations, further impacting both the precision of infrared temperature measurements and the subsequent defect identification.
2. Idealization Deviations of Material and Model Parameters: In this study, both experimental and simulation models assumed that material property parameters are uniformly distributed. However, in practice, discrepancies exist between the actual thermal conductivities—such as the uniformity of heat conduction in formwork materials and concrete—and those assumed in the models. These differences can lead to errors in predicting the thermal contrast (Δ*T*). In particular, the presence of air gaps between the formwork and concrete may introduce additional thermal resistance, resulting in deviations between the predicted Δ*T* peak and the actual condition.
3. Limitations in Dynamic Equipment Detection: The thermal sensitivity of infrared cameras poses challenges for detecting small-scale defects within thicker formworks. Moreover, the operational complexity of infrared cameras limits their practical application on concrete pouring sites.

To address these challenges, future research should focus on the following directions:

1. Multi-Physical Field Coupled Modeling: Future studies should integrate complex experimental conditions—including ambient temperature, humidity, wind speed, and solar radiation intensity—to conduct experiments and simulations under multiple coupled conditions. The primary focus should be on investigating the mechanisms and performance of infrared detection of honeycomb defects when influenced by multiple factors simultaneously.
2. Refined Characterization of Materials and Interfaces: Based on practical engineering requirements, further research should be conducted on the material parameters and interfacial heat transfer characteristics. The goal is to elucidate the influence of material properties and interfacial heat transfer on the infrared detection of concrete honeycomb defects, thereby achieving more accurate prediction outcomes.
3. Upgrading Intelligent Detection Systems: Develop an unmanned aerial vehicle (UAV) platform equipped with a high-frame-rate infrared module. By integrating image enhancement techniques and deep learning-based image fusion methods, the signal-to-noise ratio of the thermal contrast (Δ*T*) can be improved, thereby enhancing the precision and reliability of infrared detection.

## 5. Conclusions

This study investigated honeycomb defects in concrete using infrared thermography during the hydration heat process. Finite element modeling (FEM) was employed to simulate the thermal conduction of concrete containing honeycomb defects, exploring the feasibility of defect detection via the heat generated during hydration. Infrared testing verified the accuracy and reliability of the model, ensuring the applicability of the simulation results. Furthermore, the study analyzed the effects of ambient temperature, formwork thickness, and defect size on defect detection. The experimental results show that ambient temperature, defect size, and formwork thickness have significant impacts on thermal contrast, with different environmental conditions and material parameter combinations leading to varying thermal contrast responses. Specifically, when the temperature difference between the ambient temperature and the initial concrete temperature is large, the peak value of thermal contrast is more pronounced. Large defects generate significantly higher thermal contrast than small defects, and thinner formworks provide higher thermal contrast, which is beneficial for defect detection. By analyzing the temporal evolution characteristics of thermal contrast, the optimal time window for infrared detection was determined to be between 35 and 60 min after concrete pouring. Although this study demonstrates the theoretical feasibility of infrared thermography, practical applications still face challenges such as environmental coupling effects, idealization deviations of material parameters, and limitations in equipment detection capabilities. Future research should focus on multi-physical field coupled modeling, refined characterization of material and interface heat transfer properties, and the upgrading of intelligent detection systems to enhance the accuracy and reliability of infrared detection.

## Figures and Tables

**Table 1:** Material parameters of the model.

Material	Specific Heat Capacity (J/(kg·K))	Density (kg/m^3^)	Thermal Conductivity (W/(m·K))
Wooden Board	2700	532	k(T)
Air	k(T)	k(T)	k(T)
Concrete	980	2450	0.17

**Table 2:** Working condition design.

Parameters	Group 1	Group 2	Group 3
L × D × H (mm)	400 × 400 × 1000	400 × 400 × 1000	400 × 400 × 1000
Ambient temperature Ta (°C)	10, 16, 22, 28, 34	22	16, 22
Honeycomb size (mm)	30–60	4–10, 10–30, 30–60, 200 × 200	30–60
Formwork thickness (mm)	14	14	11, 14, 17, 20

**Table 3:** Defined parameters for analysis.

Parameter Name	Parameter Explanation
*T* *c*	The initial temperature of concrete Ambient temperature Formwork surface temperature
*T* *a*	
*T* *s*	
*σ*	Standard deviation

**Table 4:** Specifications of infrared cameras.

Camera Name	FLIR A300	MAG-F6
Detector type	Uncooled microbolometer	Uncooled microbolometer
Accuracy	±2 °C or ±2%	±0.7 °C or ±0.7%
Resolution	320 × 240 pixels	640 × 480 pixels
Spectral range	7.5–13 µm	7.5–13 µm

**Table 5:** Concrete mix proportion (1 m^3^).

Cement (kg)	Fly Ash (kg)	Silica Ash (kg)	Sweller (kg)	Sand (kg)	Aggregate (kg)	Water (kg)	Polycarboxylate Superplasticizer (kg)
411	89	35	59	968	731	143	7.08

## Data Availability

The raw data supporting the conclusions of this article will be made available by the authors on request.
